# Comment on ‘Childhood leukaemia and socioeconomic status in England and Wales 1976–2005: evidence of higher incidence in relatively affluent communities persists over time’

**DOI:** 10.1038/bjc.2012.179

**Published:** 2012-06-12

**Authors:** A Smith, T Lightfoot, E Roman

**Affiliations:** 1 Epidemiology & Genetics Unit, Department of Health Sciences, University of York, York YO10 5DD, UK

**Sir,**

Kroll *et al* (2011) recently reported their findings from an ecological analysis examining paediatric leukaemia registration rates and area-based deprivation in England and Wales. Much to our surprise, selective subgroup findings from the United Kingdom Childhood Cancer Study (www.UKCCS.org) were presented in support of their observations – in our view inappropriately so (Smith *et al*, 2006). Our comprehensive analysis, published in 2006, stated ‘No differences in area-based measures of deprivation were observed between cases and controls at time of diagnosis, either for all cancers combined (*N*=4430; OR=1.00, 95% CI=0.98–1.01) or for acute lymphoblastic leukaemia (ALL) alone (*N*=1578, OR=0.99, 95% CI=0.99, 95% CI=0.96–1.01). Findings were similar at time of birth (all cancers OR=0.99, 95% CI=0.98–1.01); (ALL OR=0.98, 95% CI=0.96–1.00). In addition, no case-control differences were observed when an individual-based measure of SES – social class – based on father’s occupation at time of birth was used’. These results were based on all cases diagnosed across the country as a whole and all randomly selected ‘first-choice’ controls – regardless of whether or not their parents were interviewed in the main study.

Kroll *et al* (2011) adopted an analytical approach different from ours, concentrating on trend tests and differences between the top and bottom quintile deprivation categories. Leaving aside the merits of the different approaches, our findings and conclusions were not affected by the analytical method used – a fact that is evident from the data presented in our paper. A major strength of the UKCCS is that it can be used to examine the potential impact of various biases – which we discussed at length in our original paper. Given this, we were very surprised to see that instead of quoting from our main results table, as many others have done (Adam *et al*, 2008; CRUK, 2011), Kroll *et al* presented effect measures from the table that excluded the 595 cases whose parents were not interviewed in the main study – despite the fact that we stated that ‘the 595 non-interviewed cases tended to live in more-deprived areas’. Clearly, this is the reason why, in our data, the risk estimate for ALL for the most deprived quintile relative to the most affluent in that specific table was 0.76 (0.61–0.95). The odds ratios that should have been quoted are in the table for all 1578 children: 1.0 (reference), 1.10 (0.93–1.30), 0.96 (0.81–1.14), 1.02 (0.86–1.22), and 0.90 (0.97–1.07) for deprivation quintiles 1–5 (Smith *et al*, 2006). Co-incidentally, within the same edition as the paper by Kroll *et al* (2011), we presented data from another of our population-based studies that also found no evidence of an association between leukaemia incidence (paediatric and/or adult) and material deprivation (Smith *et al*, 2011).

In summary, we stand by our original conclusion that the systematic variations with socioeconomic reported in some ecological studies reflect differences in case notification – not differences in underlying disease occurrence (Smith *et al*, 2006). However, this does not mean that we are unaware of the importance of socioeconomic factors in the disease process. Indeed, using the same UKCCS data set we recently showed that although deprivation is not associated with ALL development, it is related to disease outcome – children in lower deprivation quintiles have significantly higher mortality than those in more affluent groups (Lightfoot *et al*, 2012).
